# Impact of Chronic Obstructive Pulmonary Disease on Atrial Fibrillation Ablation Outcomes According to the National Readmission Database

**DOI:** 10.19102/icrm.2022.130806

**Published:** 2022-08-15

**Authors:** Ahmed M. Maraey, Muhammad Haisum Maqsood, Mahmoud Khalil, Ahmed Hashim, Ahmed M. Elzanaty, Hadeer R. Elsharnoby, Eman Elsheikh, Lamiaa Elbatanony, Kenneth Ong, Paul Chacko

**Affiliations:** ^1^Department of Internal Medicine, CHI St. Alexius Health, Bismarck, ND, USA; ^2^Department of Internal Medicine, University of North Dakota, Bismarck, ND, USA; ^3^Department of Internal Medicine, Lincoln Medical Center, Bronx, NY, USA; ^4^Department of Cardiology, Tanta University Faculty of Medicine, Tanta, Egypt; ^5^Ain Shams University, Cairo, Egypt; ^6^Department of Cardiovascular Medicine, University of Toledo, Toledo, OH, USA; ^7^Tanta University Faculty of Medicine, Tanta, Egypt; ^8^Department of Cardiovascular Medicine, Lincoln Medical Center, Bronx, NY, USA

**Keywords:** Atrial fibrillation, catheter ablation, COPD, mortality, outcome, readmission

## Abstract

Chronic obstructive pulmonary disease (COPD) is a risk factor for the development of atrial fibrillation (AF). There is a paucity of contemporary data studying the association between COPD and outcomes of AF ablation. The objective of this study was to investigate the impact of COPD on AF ablation outcomes using a large nationwide database. This study was a retrospective analysis of the National Readmission Database for the years 2016–2018 and included patients admitted with a diagnosis of AF who underwent catheter ablation. Admissions were stratified according to COPD diagnosis using International Classification of Diseases, 10th Revision, Clinical Modification codes. Multivariate, linear, Cox, and logistic regressions were performed to study the impact of COPD on AF ablation. A total of 18,224 admissions (mean age, 68 years; standard deviation, 10 years) were included, of whom 3,494 (19%) had a diagnosis of COPD. The COPD group was older (72 ± 8 vs. 67 ± 11 years, *P* < .001) and more likely to have congestive heart failure (73% vs. 44%, *P* < .001) and renal failure (31% vs. 17%, *P* < .001). COPD was associated with an increased risk of readmission (adjusted hazard ratio [aHR], 1.40; 95% confidence interval [CI], 1.26–1.56; *P* < .001) and all-cause in-hospital mortality (adjusted odds ratio, 2.83; 95% CI, 1.74–4.60; *P* < .001). However, COPD was not associated with an increased risk of readmission due to recurrent AF (aHR, 0.97; 95% CI, 0.75–1.27; *P* = .844) or the need for re-ablation (aHR, 0.85; 95% CI, 0.44–1.65; *P* = .639), respectively. In conclusion, COPD was not associated with an increased risk of recurrent AF after ablation despite higher periprocedural risks. The present study contributes to a better understanding of this high-risk subgroup of patients undergoing AF ablation.

## Introduction

Chronic obstructive pulmonary disease (COPD) is a disease characterized by airflow limitations due to small airway disease (obstructive bronchiolitis) and parenchymal destruction (emphysema), leading to expiratory flow limitation and hyperinflation. COPD continues to pose a growing contribution to the overall prevalence of non-communicable diseases.^1^ It was estimated that the global prevalence of COPD was 11.7% by 2010.^2^ In addition, COPD is usually associated with other comorbidities, especially atrial fibrillation (AF), the most common sustained arrhythmia affecting 33.5 million people globally.^3,4^ COPD is present in about 23% of patients diagnosed with AF.^5–7^ AF development is directly associated with the frequency of COPD exacerbation.^8^ The co-presence of both COPD and AF represents an extra challenge that requires tailoring any medical interventions to minimize any potential collateral damage.

In this study, we have investigated the potential effect of COPD on the outcomes of AF ablation.

## Methods

### Data source

This was a retrospective cohort study performed using the Agency for Healthcare Research and Quality’s Healthcare Cost and Utilization Project (HCUP) Nationwide Readmission Database (NRD) for the years 2016–2018.^9^ The NRD is the largest publicly available all-payer inpatient health care readmission database in the United States. Data in the NRD are drawn from HCUP state inpatient databases containing verified patient linkage numbers that can be used to track a person across hospitals within a state while adhering to strict privacy guidelines. It provides longitudinal information about patients’ initial hospitalization and subsequent readmissions in a calendar year. It contains a weighted sample of hospitalizations in the United States, and this can be used to directly derive national estimates of various hospitalizations. National estimates were produced using sampling weights provided by the sponsor. All values presented are weighted estimates.

### Study population

Our study population included admissions with a diagnosis of AF who underwent catheter ablation. To define the admissions with AF who underwent catheter ablation, we used the International Classification of Diseases, 10th Revision (ICD-10), Clinical Modification, codes for AF who underwent catheter ablation (ICD-10 Procedure Coding System codes [02573ZZ, 025S3ZZ, 025T3ZZ, 02583ZZ]) **(Supplementary Table 1)**. Admissions with a diagnosis of other cardiac arrhythmias for which an ablation procedure may be required were excluded. Arrhythmias excluded were supraventricular tachycardia, ventricular tachycardia, atrial flutter, ventricular premature beats, pre-excitation syndrome, atrioventricular (AV) nodal tachycardia, and atrial tachycardia. Also, to avoid inclusion of patients undergoing AV junction ablation, we excluded admissions with codes indicating prior or current implantation of a pacemaker or an implantable cardioverter-defibrillator. This approach for the identification of admissions with AF and catheter ablation has been previously described and used by other investigators.^10^ In NRD, patient identifiers cannot be linked across years. Hence, index admissions on July 1 or beyond were excluded to allow 6 months of follow-up. Other exclusion criteria were admissions with age <18 years and those with missing data.

In accordance with the HCUP data use agreement, we excluded reporting any variable containing a small number of observations (≤10) that could pose a risk of person identification or data privacy violation. This study was exempted from approval by the institutional review board because of the anonymized and de-identified data in the NRD.

### Study outcomes

Primary outcomes were 180-day readmission with recurrent AF, AF re-ablation, and all-cause readmissions after index admissions for AF ablation.

Secondary outcomes were in-hospital mortality, iatrogenic cardiac complication, postprocedural bleeding, stroke, transient ischemic attack (TIA), acute myocardial infarction, cardiac tamponade, acute kidney injury (AKI), and acute heart failure. Index admission was defined as the first admission with the primary diagnosis of AF without prior admission in a 180-day period. A readmission was defined as the first readmission within 180 days of admission.

### Statistical analysis

Continuous variables were compared using Student’s *t* test, and categorical variables were compared using the chi-squared test. Multivariate regression analyses were used to study the association between COPD and AF ablation outcomes and to adjust for confounders: age; sex; median income; primary insurance; hospital bed size; hospital teaching status; and chronic comorbidities such as hypertension, diabetes mellitus, congestive heart failure, valvular heart disease, peripheral vascular disease, obesity, liver disease, history of myocardial infarction, history of percutaneous coronary intervention, and history of coronary artery bypass graft. Logistic regression was used for dichotomous in-hospital outcomes, and linear regression was used for continuous outcomes. Cox proportional hazards regression analysis was used in readmission outcomes. In-hospital mortality admissions were excluded from readmission analysis. Continuous data were presented as mean and standard deviation (SD) values. All *P* values were 2-sided, with 0.05 as the threshold for statistical significance. Data analysis was performed using STATA 17 (StataCorp, College Station, TX, USA).

## Results

### Baseline characteristics

Our cohort included 18,224 AF ablation admissions (mean age, 68 ± 10 years) between January and June of 2016–2018, of whom 3,494 (19%) admissions had a diagnosis of COPD. The mean age was different between the COPD group and the non-COPD group (72 ± 8 and 67 ± 11 years, *P* < .001, respectively). COPD admissions were likely to be women (46% vs. 43%, *P* = .02). Medicare was the primary insurance in the cohort; however, COPD admissions were more likely to have Medicare insurance (81% vs. 60%, *P* < .001). AF ablation was less likely to be done in a teaching hospital among the COPD group (80% vs. 95%, *P* < .001). COPD admissions were more likely to have congestive heart failure (73% vs. 44%, *P* < .001), peripheral vascular disorder (21% vs. 12%, *P* < .001), renal failure (31% vs. 17%, *P* < .001), chronic respiratory failure (18% vs. 1%, *P* < .001), and hypothyroidism (20% vs. 16%, *P* < .001). Other baseline characteristics stratified according to the presence or absence of COPD are included in **Table 1**.

### Outcomes

A total of 5,209 readmissions within 180 days occurred after AF ablation, of whom 1,505 (29%) carried a diagnosis of COPD. COPD was associated with an increased risk of readmission after adjustment (adjusted hazard ratio (aHR), 1.40; 95% confidence interval [CI], 1.26–1.56; *P* < .001).

**Figure 1:**
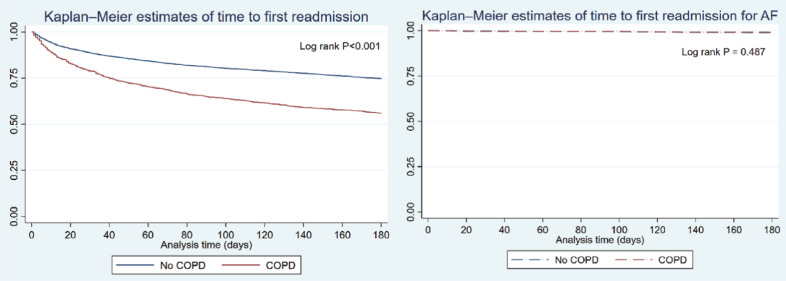
Kaplan–Meier estimates comparing time to first all-cause readmission and first atrial fibrillation readmission in the chronic obstructive pulmonary disorder (COPD) group versus non-COPD group. *Abbreviations:* AF, atrial fibrillation; COPD, chronic obstructive pulmonary disease.

There were a total of 746 readmissions with AF, of whom 151 underwent re-ablation. COPD was not associated with an increased risk of readmission due to recurrent AF (aHR, 0.97; 95% CI, 0.75–1.27; *P* = .844) or the need for re-ablation (aHR, 0.85; 95% CI, 0.44–1.65; *P* = .639) **(Figure 1)**.

In-hospital mortality occurred in 168 (1%) admissions. COPD was associated with an increased in-hospital mortality. The adjusted odds ratio (aOR) was 2.83 (95% CI, 1.74–4.60; *P* < .001).

**Figure 2:**
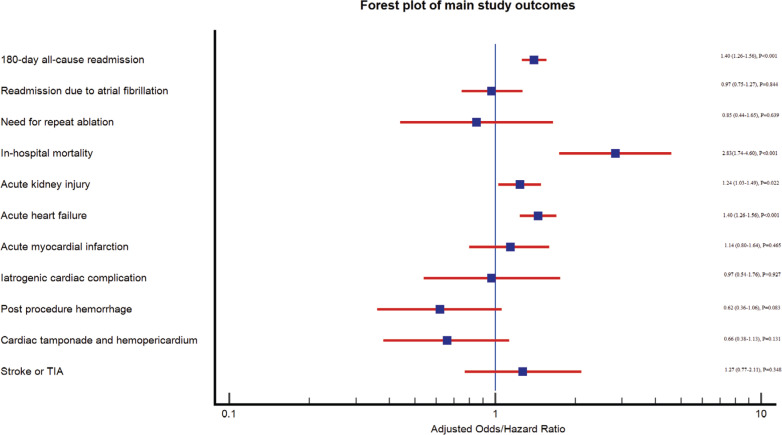
Forest plot of main study outcomes. *Abbreviation:* TIA, transient ischemic attack.

**Figure 3:**
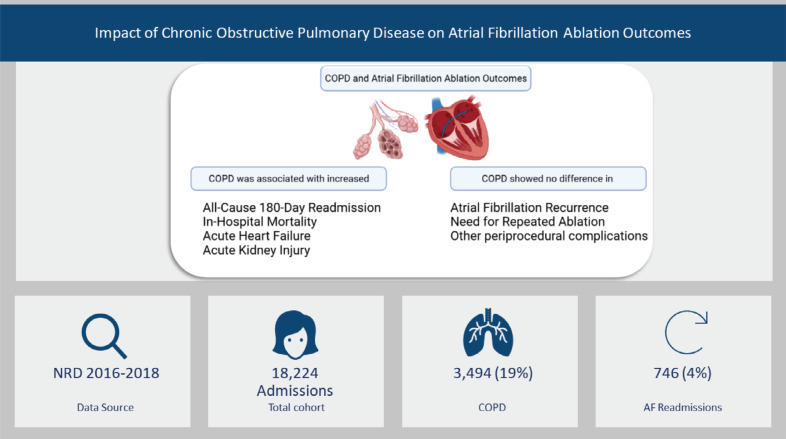
Study summary. *Abbreviations:* AF, atrial fibrillation; COPD, chronic obstructive pulmonary disease; NRD, Nationwide Readmission Database.

In terms of periprocedural complications, COPD was not associated with an increased odds of postprocedural hemorrhage (aOR, 0.62; 95% CI, 0.36–1.06; *P* = .083), iatrogenic cardiac complications (aOR, 0.97; 95% CI, 0.54–1.76; *P* = .927), cardiac tamponade (aOR, 0.66; 95% CI, 0.38–1.13; *P* = .131), acute myocardial infarction (aOR, 1.14; 95% CI, 0.80–1.64; *P* = .465), or stroke (aOR, 1.27; 95% CI, 0.77–2.11; *P* = .348). However, it was associated with an increased risk of acute heart failure (aOR, 1.40; 95% CI, 1.26–1.56; *P* < .001) and AKI (aOR, 1.24; 95% CI, 1.03–1.49; *P* = .022) **(Figures 2 and 3 and Table 2)**.

## Discussion

This nationally representative analysis of 18,224 AF admissions who underwent ablation, using NRD data from the years 2016–2018, found no difference in 180-day readmission due to AF or re-ablation despite the higher risk of all-cause readmissions and some periprocedural complications, such as acute heart failure, in-hospital mortality, and AKI, in the COPD group compared to the non-COPD group. Other periprocedural complications, such as acute myocardial infarction, iatrogenic cardiac complications, postprocedural hemorrhage, cardiac tamponade and hemopericardium, and stroke/TIA, were not different between the 2 groups.

It is well known that COPD has been associated with an increased incidence of AF,^11–13^ which is attributed to increased arrhythmogenicity from hypoxia,^14^ hypercapnia,^15^ pulmonary hypertension,^16^ ventricular remodeling,^17^ inflammation,^18^ and respiratory drug use.^19^ At a biochemical level, the forementioned stresses are hypothesized to induce the production of hypoxia-inducible factor 1a, vascular endothelial growth factors, and matrix metalloproteinase-9 through a cascade of reactions that ultimately leads to atrial fibrosis.^14,20–22^

In 2011, a retrospective study on AF ablation from South Korea from 2000–2009 sparked a huge interest in AF ablation in chronic lung disease patients.^23^ They did not only find out that chronic lung disease changes the pulmonary vein anatomy and makes it more arrhythmogenic but also reported that non-pulmonary vein foci–related AF was more common in chronic lung disease patients. In 2015, a review by Goudis on the relationship between COPD and AF highlighted the need for studies on AF ablation outcomes in COPD patients.^24^ Similarly, the abovementioned gap was reinforced in recent reviews on AF, COPD, and pulmonary hypertension.^25,26^

So, this nationally representative cohort of American adult patients is the first large study to evaluate the outcomes of AF ablation in patients with COPD. This is also the first study of its kind, which evaluated 180-day readmission and other procedure-related complications of AF ablation.

A national cohort analysis of years 2010–2014, which evaluated causes and predictors of readmission (30-day and 90-day readmission) post-ablation, found that patients with chronic pulmonary disease have a higher risk of readmission and COPD is an independent predictor of readmission.^10^ The results of that analysis align with our analysis, which showed a significantly higher risk of readmission in COPD patients.

AF has been challenging to control in patients with COPD. Non-COPD status was one of the independent predictors of successful electrical cardioversion at 1 year of follow-up in the Euro Heart Survey (*P* = .003).^27^ A prospective study by Gu et al. on 550 consecutive patients with symptomatic and medication-refractory AF with first catheter ablation found a significantly greater recurrence of atrial tachyarrhythmias in patients with COPD versus patients without COPD (44% vs. 28.6%, *P* = .016) with a mean follow-up of 31.4 months.^28^ In contrast, our analysis with a shorter follow-up duration (6 months) did not find differences between the 2 arms (COPD vs. non-COPD) in readmission with AF or the need for repeat ablation.

Our study demonstrated that post-ablation in-hospital mortality in patients was higher in the COPD group (2% vs. 1%, *P* < .001). Our results are comparable to those of a multicenter analysis of AF patients from 13 hospitals in China from 2008–2011. The study found that the prevalence of COPD was significantly higher in the mortality group than the survival group (21.4% vs. 10.1%, *P* ≤ .01).^29^

In the present study, we observed a higher risk of acute congestive heart failure and AKI in the COPD group. New-onset congestive heart failure was reported following AF catheter ablation by Tan et al.^30^ Congestive heart failure was found to be an independent predictor of worsening renal function in a prospective study done by Kawaji et al. on 791 non-dialysis patients who underwent AF ablation.^31^ Due to the lack of patient-level data in the administrative database, such as the degree of ejection fraction reduction and the level of kidney impairment, we could not further investigate the increased risk of acute heart failure and AKI in COPD patients. We hypothesize that the difference was likely due to the higher prevalence of chronic heart failure and chronic kidney disease in the COPD arm and the strong correlation between COPD and congestive heart failure.

COPD has been associated with a greater risk of arteriovascular events, including ischemic stroke, cerebrovascular events, TIAs, and myocardial infarctions.^32–34^ Similarly, COPD patients are more likely to have peripheral vascular diseases and atherosclerosis.^35^ The present study did not find a difference in outcomes of in-hospital occurrence of acute myocardial infarction and ischemic stroke/TIA between the 2 groups segregated based on COPD status. However, our study evaluated these outcomes if they happened during the index admission. It would be interesting to see future studies on post-ablation vascular complications in the 2 groups.

### Limitations

This study should be interpreted in light of some important limitations. First, it is an observational study subject to confounding bias despite statistical adjustment. Second, the severity of COPD and other comorbidities, anti-arrhythmic medication use, and medication compliance could not be assessed due to the administrative nature of the database, which is also subject to coding errors. Third, a real-world all-payer, nationally representative NRD cohort could not include all admissions in the United States, so it might over- or underestimate the overall difference in outcomes. Additionally, AF recurrence without admission was not captured. Fourth, the follow-up period was relatively short as admissions cannot be linked across years. Longer-term follow-up will be an area for future research.

## Conclusion

Despite being a risk factor for the development of AF, COPD was not associated with an increased risk of AF recurrence after the ablation procedure. However, it was associated with increased AF ablation periprocedural complications. The present study contributes to a better understanding of this high-risk subgroup of patients undergoing AF ablation. The development of a nationally valid risk stratification model and improving post-hospital care have the potential to offset some of the risks inherent to ablating AF in COPD patients.

## Figures and Tables

**Table 1:** Baseline Characteristics Stratified According to the Presence or Absence of COPD

Characteristics	AF Ablation		*P* Value
	No COPD (n = 14,523)	COPD (n = 3,432)	
**Female sex**	6,318 (43%)	1,881 (46%)	.018
**Mean age in years (SD)**	72 (±8)	67 (±11)	<.001
**Median household income quartile for zip code per percentile**			<.001
<25^th^	3,089 (21%)	944 (28%)	
25^th^–50^th^	3,687 (25%)	987 (29%)	
50^th^–75^th^	3,735 (26%)	877 (26%)	
;75^th^	4,011 (28%)	623 (18%)	
**Insurance**			<.001
Medicare	8,854 (60%)	2,815 (81%)	
Medicaid	623 (4%)	187 (5%)	
Private	4,804 (33%)	408 (12%)	
Self-pay	109 (1%)	22 (1%)	
Other/no charge	334 (2%)	60 (2%)	
**Hospital bed size**			.196
Small	899 (6%)	263 (8%)	
Medium	3,956 (27%)	972 (25%)	
Large	9,875 (67%)	2,259 (65%)	
**Teaching hospital**	12,493 (85%)	2,802 (80%)	
**Charlson Comorbidity** **Index score**			<.001
≤1	8,926 (60%)	445 (13%)	
2	2,304 (16%)	850 (24%)	
≥3	3,500 (24%)	2,198 (63%)	
**Comorbidities**			
Congestive heart failure	6,492 (44%)	2,563 (73%)	<.001
Valvular heart disease	3,257 (22%)	950 (27%)	<.001
Peripheral vascular disease	1,803 (12%)	748 (21%)	<.001
Pulmonary circulatory disorder	1,110 (8%)	590 (17%)	<.001
Uncomplicated hypertension	6,617 (45%)	1,147 (33%)	<.001
Complicated hypertension	4,570 (31%)	1916 (55%)	<.001
History of myocardial infarction	1,052 (7%)	462 (13%)	<.001
History of PCI	1,327 (9%)	503 (14%)	<.001
History of CABG	927 (6%)	370 (11%)	<.001
History of stroke	1,109 (8%)	327 (9%)	.014
Renal failure	2,571 (17%)	1,081 (31%)	<.001
Obesity	3,484 (24%)	990 (28%)	<.001
Hypothyroidism	2,338 (16%)	707 (20%)	<.001
Dyslipidemia	6,553 (44%)	1,750 (50%)	<.001
Diabetes without complications	2,289 (16%)	610 (17%)	.045
Diabetes with complications	1,456 (10%)	682 (20%)	<.001
Liver disease	349 (2%)	156 (4%)	<.001
Alcohol abuse	327 (2%)	167 (5%)	<.001
Drug abuse	132 (1%)	67 (2%)	<.001

*Abbreviations:* AF, atrial fibrillation; CABG, coronary artery bypass graft; COPD, chronic obstructive pulmonary disease; PCI, percutaneous coronary intervention; SD, standard deviation.

**Table 2:** Study Outcomes

Outcome	AF Ablation		*P* Value
	No COPD (n = 14,523)	COPD (n = 3,432)	
**In-hospital mortality (n)**	88 (1%)	80 (2%)	<.001
Adjusted odds ratio	Reference	2.83 (95% CI, 1.74–4.60)	<.001
**Acute kidney injury (n)**	1,711 (12%)	809 (23%)	<.001
Adjusted odds ratio	Reference	1.24 (95% CI, 1.03–1.49)	.022
**Acute myocardial infarction**	250 (2%)	99 (3%)	.002
Adjusted odds ratio	Reference	1.14 (95% CI, 0.8–1.6)	.47
**Iatrogenic cardiac complication**	164 (1%)	36 (1%)	.78
Adjusted odds ratio	Reference	0.97 (95% CI, 0.54–1.76)	.93
**Postprocedural** **hemorrhage**	230 (2%)	34 (1%)	.062
Adjusted odds ratio	Reference	0.62 (95% CI, 0.36–1.06)	.083
**Cardiac tamponade and hemopericardium**	219 (1%)	28 (1%)	.022
Adjusted odds ratio	Reference	0.66 (95% CI, 0.38–1.13)	.131
**Stroke or TIA**	147 (1%)	47 (1%)	.204
Adjusted odds ratio	Reference	1.27 (95% CI, 0.77–2.11)	.35
**Acute heart failure**	2,735 (19%)	1,466 (42%)	<.001
Adjusted odds ratio	Reference	1.45 (95% CI, 1.24–1.70)	<.001
**180-day all-cause readmission**	3,700 (25%)	1,505 (44%)	<.001
Adjusted hazard ratio	Reference	1.40 (95% CI, 1.26–1.56)	<.001
**Readmission due to atrial fibrillation**	627 (4%)	119 (3%)	.12
Adjusted hazard ratio	Reference	0.97 (95% CI, 0.75–1.27)	.844
**Need for repeat ablation**	131 (1%)	20 (1%)	.18
Adjusted hazard ratio	Reference	0.85 (95% CI, 0.44–1.65)	.639

*Abbreviations:* AF, atrial fibrillation; CI, confidence interval; COPD, chronic obstructive pulmonary disease; TIA, transient ischemic attack.

**Supplementary Table 1:** International Classification of Diseases, Tenth Edition, Clinical Modification (ICD-10-CM) Codes Used to Identify Key Variables

**Atrial Fibrillation**	I48, I48.0, I48.1, I48.11, I48.19, I48.2, I48.20, I48.21, I48.91
**Catheter ablation**	02573ZZ, 025S3ZZ, 025T3ZZ, 02583ZZ
**Chronic obstructive pulmonary disease**	J44, J44.0, J44.1, J44.9, J41, J41.0, J41.1, J41.8, J42, J43.0, J43.1, J43.2, J43.8, J43.9
**Premature ventricular complexes**	I49.3
**Supraventricular tachycardia**	I47.1
**Ventricular premature depolarization**	I49.3
**Pre-excitation syndrome**	I45.6
**Presence of pacemaker**	Z95.0
**Paroxysmal tachycardia**	I47.9
**Atrial flutter**	I489.2, I48.3, I48.4
**Postprocedural hemorrhage**	I97.610, I97.618, I97.620, I97.638, J95.830, K91.841, K91.871
**Iatrogenic cardiac complications**	I97.790, I97.88, I97.89
**Hemopericardium**	I31.2
**Cardiac tamponade**	I31.4
**Acute myocardial infarction**	I21.4, I21.9, I21.3, I21.09, I21.19, I21.02, I21.21, I21.01, I21.11, I21.29
**Uncomplicated hypertension**	I10.x
**Complicated hypertension**	I11.x-I13.x, I15.x
**Valvular heart disease**	A52.0, I05.x - I08.x, I09.1, I09.8, I34.x - I39.x, Q23.0 - Q23.3, Z95.2 - Z95.4
**Pulmonary circulation disorders**	I26.x, I27.x, I28.0, I28.8, I28.9
**Peripheral vascular disorders**	I70.x, I71.x, I72.x, I73.1, I73.8, I73.9, I77.1, I77.7, I79.0, I79.1, I79.8, I79.2, K55.1, K55.8, K55.9, Z95.8, Z95.9
**Uncomplicated diabetes mellitus**	E08.9, E09.9, E10.9, E11.9, E13.9
**Complicated diabetes mellitus**	E08.2-E08.8, E09.x, E10.2 - E10.8, E11.2 - E11.8, E12.2 - E12.8, E13.2 - E13.8
**Hypothyroidism**	E00.x - E03.x
**Obesity**	E66.x, Z68.3, Z68.4, Z68.5
**Liver disease**	B18.x, I85.x, K70.x, K71.1, K71.3 - K71.5, K71.7, K72.x - K74.x, K75.4, K75.8, K76.0, K76.2 - K76.9, Z94.4
**Obesity**	E66.x, Z68.3, Z68.4, Z68.5
**Deficiency anemia**	D501, D50.8, D50.9, D51.x - D53.x, D63.1, D63.8
**Alcohol abuse**	F10, E52, G62.1, I42.6, K29.2, K70.0, K70.3, K70.9, T51.x, Z71.4
**Drug abuse**	F11.x - F16.x, F18.x, F19.x, Z71.5
**Prior myocardial infarction**	I25.2
**Prior percutaneous coronary intervention**	Z98.61, Z95.5
**Prior coronary artery bypass grafting**	Z95.1
**Dyslipidemia**	E78.5
